# Supplementary material for: Are carbon emissions trading and green financial instruments synergistic? -Comprehensive quantitative research based on content analysis
Source: PLoS One. 2024 Mar 7;19(3):e0298601. doi: 10.1371/journal.pone.0298601 (PMC10919601; doi:10.1371/journal.pone.0298601)
Supplement: S1 Data — (ZIP) [file pone.0298601.s001.zip › Data/地级市Z_F工作报告.zip!/║╙─╧/╨┬╧τ╩╨/└┤╫╘╣1⁄2╓┌║┼í╛┬φ┐╦╩2╛▌═°í┐_2019.docx]

各位代表：

　　现在，我代表市人民政府，向大会报告工作，请予审议，并请各位政协委员和列席人士提出意见。

　　一、2018年工作回顾

　　2018年是贯彻党的十九大精神开局之年，也是本届政府起步之年。全市人民以习近平新时代中国特色社会主义思想为指导，深入贯彻落实党的十九大和习近平总书记视察指导河南时的重要讲话精神，在省委、省政府和市委的坚强领导下，坚持稳中求进工作总基调，以新发展理念为引领，以供给侧结构性改革为主线，狠抓工作落实，较好完成了市十三届人大一次会议确定的年度主要目标任务。

　　——发展态势持续向好。完成地区生产总值2526.6亿元，总量保持在全省第6位，增长7.1%。一般公共预算收入172.7亿元，总量位居全省第4位，增长8.6%；税占比72.8%，高于全省平均水平2.2个百分点。固定资产投资增长10.1%，高于全省平均水平2个百分点；工业投资增长24.4%，位居全省第1位，高于全省平均水平22.4个百分点。规模以上工业增加值增长8.2%，高于全省平均水平1个百分点；利润总额增长43.6%，位居全省第2位，高于全省平均水平19.1个百分点。社会消费品零售总额增长10.5%，高于全省平均水平0.2个百分点。经济指标匹配性较好。

　　——发展新动能快速成长。深化“放管服”改革，审批时限压缩三分之二；新登记市场主体7.2万户，增长34.6%。引进境外资金11.4亿美元、省外资金675亿元，分别位居全省第3位和第5位；外贸进出口79.6亿元，增长14.6%；成功举办第一届高校院所（河南）科技成果博览会，签约项目140个、总投资1285.8亿元。新乡高新区在国家高新区综合排名84位，提升8个位次；产业升级和结构优化能力提升25个位次。改革开放创新不断释放出新的发展活力。

　　——发展势能加快积蓄。“六大专项”“六大体系”建设加快推进。编制完成生态城、沿黄生态经济带总体规划及部分专项规划。凤泉湖开工建设。获批总规模84万亩、投资74亿元的国家储备林建设项目。围绕动力电池与新能源汽车、大数据、机器人、生命科学和生物技术及石墨烯等新型材料领域，在先进制造业专业园区实施“一台两器”项目44个、制造业项目87个，完成投资93.4亿元。高质量发展迈出新步伐。

　　——发展基础不断夯实。三大攻坚战稳步推进，非法集资案件兑付16起，结案销号11起；新飞电器重整成功，实现涅槃重生；杜绝了较大以上事故发生。5.39万贫困群众稳定脱贫，封丘县、原阳县有望脱贫摘帽；农村居民人均可支配收入14939元，增长8.5%，高于城镇居民人均可支配收入增速0.8个百分点。是全省完成PM10、PM2.5治理任务的6个地市之一，优良天数增加4天，提前消除劣五类水体。常住人口城镇化率达53.4%，提高1.45个百分点。决胜全面小康的基础更加坚实。

　　一年来，我们主要做了以下工作：（一）狠抓关键环节，稳定经济运行。帮助企业解决难题。坚持各级领导分包和企业服务周例会制度，为新航集团、科隆集团等81家企业解决不动产手续办理、融资等突出问题100余个。降低企业运营成本。出台52条降成本措施，减轻企业负担30亿元以上。为1.9万户小微企业减免税收1.5亿元，减免税额增长81.6%。小微企业贷款余额较年初增加27亿元，平均利率下降1.3个百分点。扩大有效投资。在建项目1163个，增加68个，项目增量位居全省第3位；实施亿元以上项目320个，增加69个，新松机器人产业园、鲁花食品产业园等项目开工，平煤国能锂电一期、中纺院绿色纤维二期等项目投产，豫氢动力首台45千瓦质子交换膜燃料电池堆组装下线。强化要素保障。获批建设用地2.1万亩，收储土地1.3万亩。各项贷款余额增速连续15个月保持在10%以上，企业贷款较年初增加73亿元，同比多增69亿元。完成河南（新乡）氢能等3支基金注册。

　　（二）优化经济结构，提升发展质量。发展先进制造业。先进制造业专业园区实现主营业务收入259亿元，增长29.7%。实施“三大改造”重点项目442个，总投资567亿元。战略性新兴产业投资增长485.6%，占工业投资比重提高3.2个百分点；高载能行业投资比重下降0.6个百分点。大型企业对工业增加值贡献率达52.5%。115家小微企业升入规模以上企业。发展现代农业。扛稳粮食安全重任，完成450万亩粮食生产功能区划定，优质强筋小麦种植面积增加11万亩，粮食生产能力稳步提升。国家农业科技园区正式挂牌，国家生物育种产业创新中心落户新乡。发展现代服务业。滚动实施总投资1900亿元的110个现代服务业重点项目，完成年度投资155亿元。接待国内游客人数、实现旅游收入分别增长13.9%和15.9%。第三产业增加值占GDP比重提高1.7个百分点。

　　（三）加快推进新型城镇化，促进城乡协调发展。推进百城建设提质。全市开工项目492个，完成投资121亿元。中心城区实施基础设施和生态项目213个，完成投资39.6亿元；两年时间内市区供热普及率由63%提高到95%以上，兑现了让广大市民温暖过冬的承诺。加快郑新深度融合。编制完成郑新全域深度融合发展战略规划，实施总投资3428亿元的重大项目126个。提升交通支撑能力。实施总投资560亿元的交通项目45个，完成投资33.4亿元。新晋高速、G327改建、官渡黄河大桥等项目加快推进，新日高速封丘至原阳段、沿太行高速新乡段、新延快速路、G107东移、S229快速路等项目具备全面开工条件。加快乡村振兴步伐。启动“两县十乡（镇）百村”示范工程，农村人居环境明显改善，96%的村庄建立卫生长效保洁机制，完成改厕27.7万户，建成768个示范村、1632个达标村。

　　（四）推进改革开放创新，增强发展活力。深化改革。市公交总公司等国企改革加快推进，处置国有僵尸企业136户，成为全省国企改革攻坚工作先进省辖市。列入企业开办全程网上办“零见面”改革全国试点。获嘉县完成全国农村集体产权制度改革试点任务。基本完成农商行改革。扩大开放。签约项目384个，总投资2393亿元，其中10亿元以上项目90个，总投资1455亿元。清华大学纳米空心硅产业园、三一集团装配式建筑等一批重大项目落地。建信人寿等8家金融机构落户。中欧班列（新乡号）实现每月6列常态化运营，新日韩铁海联运国际班列成功开通，新乡正加速融入“一带一路”。推动创新。继高新区之后，经开区、平原示范区、牧野电源产业园、新东产业集聚区相继完成体制机制改革。深实科技研发的手机芯片和投影仪芯片分别进入中兴通讯和小米科技的供应链。33家高新技术企业通过国家认定，新获批院士工作站4家，清华大学、同济大学、中科院、欧洲农业物理研究院等一批高校和科研院所在新乡建立创新平台。全省首家科技金融中心挂牌运营。中国（新乡）知识产权保护中心获批建设，专利授权量7183件，增长68%，专利质押融资增长60%。新增中国驰名商标3件、中国地理标志商标5件，总量均位居全省前列。

　　（五）打好三大攻坚战，守住发展底线。防范化解重大风险。处置银行业不良贷款33.6亿元，是全省三个不良贷款率下降的省辖市之一。与中原资产合作确定9家企业作为首批担保链风险化解试点。非洲猪瘟疫情处置工作得到农业农村部肯定。76个问题楼盘已彻底解决18个、基本解决42个，平原体育中心等一批“问题工程”得到妥善化解。推进精准脱贫。528个产业扶贫项目完工，贫困户产业扶持和增收措施实现全覆盖。新增扶贫小额贷款19.7亿元，户获贷率91.2%，较年初提高66个百分点。完成农村危房改造1.2万户。贫困人口大病集中救治6760人，重病兜底保障574人，均实现全覆盖。发放各类教育扶贫资金8025万元，惠及贫困学生12.4万人次。扎实开展滩区迁建和易地扶贫搬迁，77.2万平方米安置房竣工，207.7万平方米安置房主体完工，3526人迁入新居。加强污染防治。持续强化“六控”，推进污染源头防控，空气质量明显改善；加快实施“四水同治”，卫河清淤复堤完工，共产主义渠清淤加快推进，原阳堤南、东大宫等引黄口门完成改造，断面水质达标率80.2%；重点区域种植结构调整完成省定任务，凤泉区、获嘉县建成7000亩花卉苗木基地。

　　（六）着力改善民生，共享发展成果。公共财政民生支出302.9亿元，增长8.6%，占一般公共预算支出的75%。城镇新增就业7.7万人，新增农村劳动力转移就业2.5万人，城镇登记失业率3.8%。城乡低保保障标准分别提高到每人每月470元、每人每年3450元；“积分养老”模式成为全国典型。新建、改扩建公办幼儿园23所；新增城镇义务教育学校12所，新增学位2.2万个；完成农村义务教育校舍维修改造11.7万平方米；本科一批上线率高于全省5.5个百分点；河南物流职业学院开始招生，我市又新增一所高等院校。完善分级诊疗制度，基层诊疗能力不断提升；二级以上公立医院全部取消药品加成，药品价格平均降幅13.5%；妇女“两癌”筛查17.4万人次，新生儿“两病”筛查率92.5%，均超额完成年度任务；中心医院东区医院完成工程总量的90%，省人民医院平原医院开工建设，市第一人民医院通过“三甲”验收。建成基层综合性文化服务中心3120个，覆盖率100%。被教育部确定为全国青少年校园足球改革试验区。严厉打击破坏文物行为，果断处置赵长城损毁事件，制定文物保护政策措施，文化遗产保护力度持续加大。推进军民深度融合，驻新部队为地方发展作出重要贡献。民族宗教工作创新推进。深入开展扫黑除恶专项斗争，打击各类违法犯罪活动。食品药品安全监管持续加强。信访总量明显下降，社会大局保持稳定。外事、海关、侨务、统计、史志、气象、地震、社科研究等工作取得新成绩。

　　我们自觉接受人大监督、政协民主监督和社会监督，认真办理人大代表建议和政协提案。加强法治政府建设，推进依法行政，强化审计监督，持续整治“四风”，政府自身建设和治理能力不断提升。

　　各位代表，过去一年取得的成绩，是在省委、省政府和市委坚强领导下，全市人民以习近平新时代中国特色社会主义思想为指引，团结拼搏的结果，是各界人士和海内外朋友鼎力支持的结果。我代表市人民政府，向全市人民，向各位人大代表、政协委员，向各民主党派、工商联、无党派人士和各人民团体，向驻新人民解放军、武警部队官兵和中央、省驻新单位致以崇高的敬意！向所有关心支持新乡发展的海内外朋友表示衷心的感谢！

　　各位代表，这些成绩的取得，充分说明市委、市政府近年来的思路和举措是符合新乡实际的，我们确定的一系列打基础、利长远的战略布局正在逐步落地实施并发挥作用。我们选准路子。围绕把习近平新时代中国特色社会主义思想、总书记交给河南打好“四张牌”任务在新乡具体化，研判形势、把握趋势、借势驭势、突出特色，确定的“六大抓手”“六大专项”“六大体系”等部署与全省战略高度契合。我们拉高标杆。以世界眼光、国际标准、新乡特色，高起点、高水平编制完成总体城市设计、综合交通、水生态文明等10余项战略规划及专项规划，科学引领各项工作开展。我们明确抓手。在推进战略谋划、策划、规划、计划过程中，坚持政策、举措、项目、机制等一体推进，把战略谋划迅速转化为战略规划，把规划深化成项目，把项目推进具体成台账，通过机制化推进台账落实，促进了一批重大项目加快实施。我们健全机制。以制度承载机制，靠机制推动工作，在经济运行、项目建设、环境保护、脱贫攻坚等领域健全了一系列体制机制，保障了各项工作高效运转。这些被实践证明的正确思路和举措，必须持之以恒、一以贯之抓好落实。

　　各位代表，我们清醒认识到，经济社会发展还面临不少困难和问题：推进高质量发展任务艰巨。增长方式粗放、结构性矛盾突出、创新能力不强的状况尚未根本改变，县域经济总体结构不优、动能不强、水平不高，需要着力加以解决。经济下行压力加大。实体经济困难较多，民营和小微企业融资难、融资贵问题尚未有效缓解，部分企业经营更加困难。风险隐患不容忽视。经济社会矛盾交织，市场、要素、环境约束趋紧，企业经营、政府债务以及金融、社会等领域的风险隐患依然存在。民生领域还有不少短板。脱贫攻坚任务艰巨，生态环境形势依然严峻，教育、医疗、养老等公共服务还不能很好满足群众需要。政府工作存在不足。少数干部懒政怠政，一些领域腐败问题仍时有发生。我们一定直面问题、勇于担当，努力把各项工作做得更好。

　　各位代表，在新时代推进高质量发展的大潮中，前方有许多立足潮头的先行者，身旁有许多豪情满怀的同路人，后面有许多奋勇争先的急行军。我们近年来跨过了不少的沟沟坎坎，但我们仍要闯过许多的激流险滩。我们坚信，没有比人更高的山，没有比脚更长的路，只要汇聚起620万牧野儿女追梦的磅礴力量，我们就一定能够在新的征程中高歌猛进、一往无前、再创辉煌。

　　二、2019年工作安排

　　今年是新中国成立70周年，是全面建成小康社会的关键之年。政府工作要落实市委十一届九次全会要求，以习近平新时代中国特色社会主义思想为指导，全面贯彻党的十九大和十九届二中、三中全会及中央经济工作会议、省委十届八次全会精神，把学习习近平总书记参加河南代表团审议时的重要讲话和视察指导河南时的重要讲话贯通起来，统筹推进“五位一体”总体布局，协调推进“四个全面”战略布局，坚持稳中求进工作总基调，坚持以人民为中心的发展思想，坚持新发展理念，坚持推动高质量发展，坚持以供给侧结构性改革为主线，坚持深化市场化改革、扩大高水平开放，贯彻“巩固、增强、提升、畅通”八字方针，落实“六稳”要求，坚决打好打赢三大攻坚战，持续推进“四个着力”、打好“四张牌”，持续实施“五大战略”，着力突出“六大抓手”、抓好“六大专项”、构建“六大体系”，统筹推进稳增长、促改革、调结构、惠民生、防风险、保稳定工作，保持经济运行在合理区间，提振市场信心，增强人民群众获得感、幸福感、安全感，保持经济持续健康发展和社会大局稳定，为全面建成小康社会收官打下决定性基础，为中原更加出彩作出新的更大贡献，以优异成绩庆祝中华人民共和国成立70周年。

　　今年经济社会发展的主要预期目标是：地区生产总值增长7.5%左右，一般公共预算收入增长7.5%以上，税收占一般公共预算收入比重达到73%以上，规模以上工业增加值增长7.5%以上，固定资产投资增长8%以上，社会消费品零售总额增长10%，居民消费价格涨幅3%左右。创新发展取得新成果。研究与试验发展经费投入强度达到2.07%左右，力争高技术产业、战略性新兴产业增加值占规模以上工业比重分别达到10%以上和20%左右。协调发展取得新成效。常住人口城镇化率提高1.5个百分点左右，义务教育阶段大班额比例控制在8%以内，人口预期寿命持续提高。绿色发展实现新提升。单位生产总值能耗、PM10和PM2.5平均浓度、空气优良天数比例、主要污染物排放量均完成省下达目标。地表水达到或好于Ⅲ类水体比例达到14.3%以上。开放发展取得新突破。实际利用外资和省外资金均增长3%，营商环境进一步优化，在全省评价的排序中位次靠前。共享发展取得新进步。居民人均可支配收入增长8%，城镇新增就业7.5万人以上，城镇登记失业率4%以内。上述预期目标体现了新发展理念和高质量发展要求，有利于把工作重点放在推进高质量发展上来。

　　全年要重点抓好以下十个方面的工作：

　　（一）稳定经济增长，巩固稳中有进的发展态势。发展是解决一切问题的基础和关键，要牢牢扭住经济建设这个中心，落实“六稳”要求，精准施策，全力支持实体经济发展。

　　强化运行调节。加强经济运行调度和监测预警，强化要素保障，搞好企业服务，确保工业平稳运行。发挥各类投资基金的引导作用，引导社会资本参与重点领域建设和运营。土地指标、环境容量、政府扶持资金优先保障重大产业项目、重大基础设施、重点民生工程。坚决清理整治“大棚房”。稳定房地产投资，提高商品房供应品质，扩大住房消费，促进房地产市场平稳健康发展。

　　扩大有效投资。大力实施高质量发展“1583”重大项目行动计划，坚持周调度、月讲评、季检查、半年观摩、年底总评等机制，推动新上项目开工，加快在建项目进度，推进竣工项目达产。实施亿元以上项目1075个，完成投资1500亿元以上，其中，10亿元以上项目318个，完成投资770亿元以上。加快氢能产业园、新松机器人产业园、纳米空心硅产业园、新乡化纤超细旦氨纶纤维二期、心连心年产2×20万吨双氧水及产业升级、孟电兴迪内高压智能成型液压装备制造等重大项目建设，力争中纺院绿色纤维三期项目建成投产。

　　发展实体经济。实体经济是国家的本钱，也是新乡经济社会发展的根基。要构建亲清新型政商关系，壮大民营企业家队伍。引导民营企业推进技术创新、产品创新、模式创新、业态创新和体制机制创新。为民营企业在创新、转型和要素保障等方面倾情倾力服务，为企业当好“保安”、做好“保姆”，帮助企业跨越“市场的冰山、融资的高山、转型的火山”。

　　（二）贯彻“八字方针”，促进经济提质增效。按照“八字方针”总要求，深化供给侧结构性改革，推动高质量发展。

　　巩固“去降补”成果。巩固煤炭、水泥、化工等行业去产能成果，推动更多产能过剩行业加快出清。确保“僵尸企业”6月底前破产清算到位、债务处置到位、人员安置到位。加大对“破、立、降”奖补支持力度，落实去产能职工安置、矿权土地、债务处置等政策，形成正向激励和优胜劣汰的政策导向。稳步推进城区企业“退城入园”。

　　增强微观主体活力。推行“多证合一”“容缺办理”等管理模式，全力压缩审批时限，让企业多用时间跑市场、少费功夫跑审批。加快诚信体系建设，完善惩戒机制，对失信违法者依法严惩、对守信守法者无事不扰。坚持政府决策执行的稳定性、连续性和一贯性，加大遗留问题的处置力度。全面开展营商环境评价，让服务对象评价政府部门。着眼“放水养鱼”，落实更大规模减税降费政策，对小微企业和科技型初创企业实施普惠性税收减免，降低社保缴费率，让企业有实打实的获得感，力争100户小微企业升为规模以上企业；为支持企业减负，各级各部门要过紧日子，大力压缩一般性支出，困难再多也一定要把这件大事办好。

　　提升产业链水平。以先进制造业专业园区为抓手，促进创新链、产业链、价值链、金融链、供应链、政策链融合，鼓励优势企业融入国内外一流企业供应链。发挥骨干企业的品牌优势，强化资源整合和低成本扩张，在产业链缺项和终端上实现突破。

　　畅通经济循环系统。着力破解生产主体和市场错配、生产和消费脱节、金融和实体失衡等难题，畅通生产、流通、分配、消费循环。加大对企业融资支持力度，帮助企业完善物权、提高抵押贷款能力；支持科隆新能源、瑞丰新材、天力锂能、卫华集团等企业在A股上市。强化产销、银企、用工、产学研对接。加快构建覆盖城乡的分拨配送物流网络。

　　（三）坚持底线思维，打好三大攻坚战。这是必须夯实的经济底板、守好的政治底线，要全力攻坚，务求必胜。

　　防范化解风险。防控金融风险。加快非法集资案件兑付，尽最大努力挽回群众损失。利用资产重组、基金并购等方式，化解担保链风险，严厉打击逃废债行为。深入推进互联网金融和网络借贷专项整治，不断优化金融生态。防控企业风险。高度关注企业股权质押、杠杆融资等潜在风险，推进市场化“债转股”，将企业负债率降到合理水平。完善无还本续贷相关配套政策，严厉惩治附加不合理贷款条件、人为拉长融资链条等行为。防控债务风险。合理控制政府债务规模，决不能难留后任、债留子孙。完善项目收益债券发行工作机制，推进生态城、棚改、收费公路、土地储备等项目收益债券发行。整合各类基金组建母基金，做大政府性基金规模。盘活土地资源、公共资产、政府性资源，提升储备土地品质和增值收益。防控安全风险。压实安全生产责任，做到隐患排查零死角、安全监管零盲点、风险管理零失控、安全失责零容忍，防范遏制各类事故发生。增强防洪抗灾能力，防范自然灾害风险。健全应急管理体系，做好突发公共事件预防处置，防范社会风险。

　　推进精准脱贫。把脱贫质量放在首位，狠抓政策落地，重点解决“两不愁、三保障”面临的突出问题，打好产业、就业创业、生态、金融扶贫“四场硬仗”，实施健康、教育、扶贫助残、易地扶贫搬迁、危房改造清零、扶贫扶志“六大行动”，推进交通扶贫、水利扶贫、电网升级和网络扶贫、环境整治“四项工程”，促进贫困群众持续稳定增收，建立脱贫正向激励和稳定脱贫长效机制。确保3.2万农村贫困群众稳定脱贫、44个贫困村退出贫困序列，确保封丘县、原阳县脱贫摘帽，完成6万人左右黄河滩区居民迁建任务。

　　抓好污染防治。保持加强生态文明建设的战略定力，决不动摇、决不松劲、决不开口子，以滚石上山的劲头抓好各项工作落实，扎实推进“四大行动”，落实“三个体系、三个机制”，严厉打击超排偷排者，严肃追究姑息纵容者，集聚更多生态财富，筑牢可持续发展的绿色根基。防治大气污染。持续抓好“六控”，加快产业结构、能源结构、运输结构、用地结构调整，推进标本兼治、依法治理、综合治理，力争空气质量排名退出全国169个重点城市后20名，还群众蓝天白云、繁星闪烁。强化政府主导和控制地位，推进矿山开采、河道采砂综合治理和资源整合，狠抓绿色矿山建设。防治水污染。落实河（湖）长制，按照“全面清河、控源截污、达标治理、清淤疏浚、开源引水”的思路，推进全域治理、系统治理、源头治理，确保出境河流水质稳定达标。上半年完成6条主要河流及一二级支流的乡镇水质自动监测站建设，确保数据监测到乡镇、责任压实到基层。将卫河、共渠治理作为重点，编制卫河全域保护规划，尽快完成共渠城区段清淤复堤。加快市区14条斜沟治理。推进人民胜利渠渠首、辛庄引黄口门改造工程和共渠新建节制闸建设。确保新乡县翟坡镇、大召营镇污水处理厂和获嘉县同盟污水处理厂扩建工程6月底前建成投运；原阳县、新乡县污水处理厂6月底前开工；对现有污水处理厂提标改造，建设尾水湿地，加强运行管理，确保污水全部纳管、提标治理、达标排放。防治土壤污染。以土壤污染综合防治先行区建设为契机，在源头预防、治理与修复等方面进行探索。建立污染地块名录及开发利用负面清单，落实监管责任，做好风险管控。

　　（四）深入推进改革开放，积聚发展新动能。以高水平开放推动高质量发展、助力全面深化改革，增强发展动能。

　　深化改革。深入推进市县机构改革，优化职能配置，提高行政效能。以敬民之心行简政之道，推进“放管服”改革，深化国家级政务服务综合标准化试点建设，推进企业登记全程电子化，推动线上“一网通办”、线下“只进一扇门”、现场“最多跑一次”，努力让企业和群众到政府办事像“网购”一样方便。推进国企改革攻坚，完成剥离国有企业办社会职能工作和全民所有制企业公司制改革，做好局委属国有企业及市公交总公司等企业公司制改革。健全国有企业法人治理结构，实现管企业向管资本的转变。持续推进投融资、水利、供销、林权等领域的改革。

　　扩大开放。加大招商力度。强力推进“一招四引”，在大数据、新能源电池、电子信息、智能家电、装配式建筑、现代纺织、机器人及现代服务业等领域再引进一批重大项目，确保新开工3亿元以上招商项目100个以上。扩大金融、商贸、文化、教育、旅游、体育、卫生、健康等领域的开放。创新招商方式。把工业用地收储、标准厂房建设、创新平台打造、产业基金设立等作为招商引资的重要抓手，把产业新城招商、园区一级开发招商作为对外开放的战略工程实施，开展“回归工程”等专题招商活动，承接发达国家和地区产业转移。坚持引进和培育并重，落实人才政策待遇，着力培养和引进高层次人才团队。打造开放平台。办好第二届高博会、新乡工业互联网高峰论坛，打造对外开放的高端平台、展示新乡形象的靓丽名片。加快建设保税物流中心、中欧班列、新日韩铁海联运国际班列“一体两翼”的国际陆港，引导制造业开展跨境贸易，加速融入“一带一路”。

　　（五）加快自创区建设，构建现代创新体系。加快建设“一区四园四集群”，提升自创区发展水平。

　　抓好“四个一批”。完善企业创新链，形成创新平台——孵化器——加速器——高新技术企业——产业园区的创新链条，把各类创新主体扶上马、送一程，新培育高新技术企业50家。新建中科院数字经济技术产业研究院、河南省电波国际联合实验室等研发机构，加强河南电池研究院研发能力建设。加快建设中国（新乡）知识产权保护中心，申建国家级知识产权运营中心。

　　加快“四园”建设。坚持科技创新、制度创新两个轮子一起转，将自创区内的政策在产业集聚区和先进制造业专业园区复制推广。推进新乡创新谷、863科技产业园、中国电波科技城、中纺院中原分院、亦创生物医药研究院、河南工业技术研究院碳纤维产业园等重大创新载体建设。发挥财政资金杆杠作用，培育各类创新创业基金，引进风投基金，加快万华高科金融园建设。推动科技与产业、科技与金融、产业与金融、军工与民用融合。

　　做强“四大集群”。发挥高新区的引领作用，围绕“动力电池抓强链、燃料电池抓建链、整车生产抓龙头”，加快科技成果转化，着力引进整车企业，用3年～5年时间打造有影响的新能源汽车产业集群。加快建设华大基因新乡中心，大力发展精准医疗和健康产业；推进万华生命科学产业城建设；推进华兰生物与省投资集团合作，发展生命科学与生物技术产业集群。推进重大关键技术“卡脖子”问题的科研攻关，确保过滤产业园建成投用，加快奈创国际石墨烯散热膜等项目建设，推动形成以军工、过滤和转向器为主的航空航天军民融合产业集群。加强大数据基础研究和核心技术攻关，推进数据产业对外经济技术合作，扩大与华为集团经济技术合作，开展联合招商，打造大数据产业集群。

　　（六）加快产业转型升级，构建现代产业体系。坚持工业立市，持续立支柱、举龙头、铸品牌、强载体，推进“六大专项”，加快改造提升传统产业，壮大主导产业，培育战略性新兴产业；高度重视“三农”工作，加快发展现代服务业。

　　加快先进制造业专业园区建设。坚持“一台两器一园”的121模式，强化“五个一”工作机制，实施重点项目75个，完成投资90亿元以上，抓好平煤国能锂电二期、豫氢动力氢燃料电池、科隆新能源电池材料等项目建设，加快中铁隧道隧道工程专用设备研究院、华兰生物单抗研究中心等平台建设。在抓好十大专业园区建设的基础上，加快中国电波科技城、新乡机器人产业园、“中国制造2025”新乡（原阳）示范区建设，力争在基础设施和产业项目建设上取得较大突破。

　　培育大企业（集团）。把现有企业发展壮大作为重大战略，扶持新航集团、卫华集团、心连心集团、白鹭投资集团、科隆集团等优势企业做强做大；支持中铁隧道、银金达等重点企业加快发展；支持康佳集团抓好新飞电器生产经营和研发能力建设，力争通过三年努力，新飞电器重新成为国内一流家电企业；支持华兰生物争创国内一流新型医药产业基地。围绕国家首批装配式建筑示范城市建设，加快天丰集团、新蒲远大、河南二建等产业基地建设。力争主营业务收入超50亿元的企业达到6家以上。

　　扎实做好“三农”工作。深入贯彻习近平总书记参加河南代表团审议时的重要讲话精神，落实中央一号文件要求，坚持农业农村优先发展，切实稳住“三农”这个基本盘，努力在农业强、农村美、农民富上取得新进展。扛稳粮食安全重任。坚持藏粮于地，落实各级政府主体责任，坚决遏制土地违法行为，坚决守牢耕地保护红线。坚持藏粮于技，加快建设国家生物育种产业创新中心、中国小麦产业研究院等创新平台，大力发展现代种业，打造优质小麦种子基地和中国种业基地；推进国家农业科技园区建设，争创国家农业高新技术产业示范区。抓好农业供给侧结构性改革。推进农业“四优四化”，优质小麦、优质花生种植面积均达到100万亩以上。延伸粮食产业链、提升价值链、打造供应链，着力构建从种子到餐桌的全产业链，实现粮食安全和现代高效农业相统一。大力实施乡村振兴战略。围绕“五个振兴”，加快推进“两县十乡（镇）百村”示范工程。编制多规合一的实用性村庄规划，把乡村振兴与“三带三区两城十镇”建设结合起来，打造具有新乡特色的振兴载体。加快推进田园综合体试点，力争建设10家以上。尽快补齐农村交通运输、农田水利、农村饮水、乡村物流、宽带网络等基础设施建设短板。抓好农村人居环境整治，开展“美丽小镇”“四美乡村”创建，改造农村户用卫生厕所16万户。全面深化农村改革。巩固和完善农村基本经营制度，深化农村土地制度和集体产权制度改革，完善农业支持保护制度，吸引返乡人才创业就业，激发乡村发展活力。

　　壮大现代服务业。加快文化、旅游、体育、康养、物流、电商、金融等产业发展，完成服务业重点项目投资180亿元以上。推进与中国动漫和四川水果侠合作，推动建设比干公园，策划、包装、引进一批现代服务业重点项目。对接国际国内一流物流企业，承接郑州物流集疏、分拨功能转移，发展公铁物流、电商物流、冷链物流、快递物流，推进LNG调峰储备-铁路接卸分销物流园、现代公铁物流园多式联运等项目建设。

　　（七）推进区域协调发展，构建现代城镇体系。以“三带三区两城十镇”为抓手，以郑新深度融合为重点，统筹区域融合、城乡融合、产业融合。

　　加快建设“三带三区两城十镇”。推进交通连接、生态对接、产业承接，促进郑新深度融合。建设郑新高新技术产业带。推进郑新产业既有垂直分工，又有水平分工，避免同质化竞争。推进封丘港北新区产业集聚区、原阳产业集聚区、平原示范区产业集聚区、“中国制造2025”新乡（原阳）示范区、获嘉华夏幸福产业新城建设，做好中原银行综合金融服务中心、平原科技城等项目前期工作。建设沿黄生态经济带。坚持“黄河岸边的山水园林、郑新融合的生态纽带、乡村振兴的重要载体、农业供给侧结构性改革的重要抓手、发展文旅康养产业的重要平台、城乡统筹发展的先行区、传承创新黄河文明的示范区”定位，开工建设原阳县和平原示范区70公里沿黄岸线、420平方公里示范段，加快中原国家植物园、中原生态绿色科创小镇建设，实施一批花木特色小镇、美丽乡村、种苗交易市场等项目，打造一批亮点，把亮点连成线路，把珍珠串成项链。建设南太行文旅康养产业带。启动编制发展规划，推进辉县市国家全域旅游示范区、国家级旅游业改革创新先行区、八里沟5A级景区创建工作，提升宝泉、关山等景区品质。建设山水林田湖草一体化生态城。按照山水林田湖草是一个生命共同体和建设公园城市的理念，坚持“北方水乡、园林经典、文化圣地、旅游天堂、创新乐园、生态新城”定位，全面启动凤泉湖湖体及景观、凤凰山生态公园等项目建设和50个村的城市更新规划，让生态塑造空间、让空间创造价值、让价值改变生活。加快路网、水网、林网、管网建设，牧野大道北延、新飞大道北延建成通车，新中大道北延、和平路北延及宝山路、双泉路开工建设。围绕文化、教育、旅游、康养、科创五大产业，加快招商引资步伐。力争生态城三年打基础、五年成规模、十年铸新城。建设延津县主题森林城。加快规划编制，稳步推进各项前期工作，确保年内开工建设。各县（市）要深入挖掘历史文化资源，彰显文化特色，强化产业支撑，谋划建设一批特色小镇。

　　深入推进百城建设提质工程和文明城市建设。按照规划、建设、管理、经营“四个高质量”要求，打造一批特色亮点项目。完完善善中中心心城城区区基基础础功功能能。。实施市政项目67项，总投资58.7亿元。抓好宏力大道东延等19条道路建设；对17个主次干道交叉口进行渠化改造；实施卫河提质示范段、夏荷公园改造等项目，新建游园、口袋公园38个；加快大东区建设步伐，提升高铁片区、定国湖片区、生态城片区、市商务中心区基础设施水平。抓好城市“双修”，提升铁西片区发展水平，建设解放大道、胜利路、人民路、平原路等示范街区，解决老旧小区水电路气暖、污水排放等问题。最大限度用好保障房建设资金和棚户区改造政策，抓好项目前期拆迁安置等工作，加快城市更新步伐。提升县城建设水平。落实“四城同建”“四水同治”“四品提升”“城市四治”等要求，实施794个百城建设提质项目，努力实现绿化、美化、亮化、净化、文化、标准化、智慧化“七化”目标。提升规划管理水平。高水平编制各项规划，将各类城市创建要求和标准融入规划，通过规划凸显城市风情、城市景观、城市记忆、城市特色；开展违法违章建筑拆除、墙体门头广告治理等专项行动，推行城市“路长制”，提升城市标准化、专业化、精细化、常态化管理水平。切实巩固文明城市创建成果，确保国家卫生城市通过复审。

　　全面振兴县域经济。深入贯彻习近平总书记关于县域治理“三起来”、乡镇工作“三结合”的重要指示，督促各县（市）、区解放思想，谋大事、创大业，激发干事创业激情。坚持产业立县、工业强县的思路不动摇，围绕先进制造业专业园区建设，每个县（市）、区至少引进1家以上高校或科研院所，设立或共建研发平台、产业研究院等创新平台。坚持把开放带动作为迅速提升发展水平的重要途径，每个县（市）、区至少引进或扩建一个投资10亿元以上、三个5亿元以上的产业项目，全力打造县域支柱产业和骨干企业。引导各县（市）、区对接中央和省市重大战略，在重大交通基础设施、生态项目等方面主动对接、自觉融入、借势发力，每个县（市）、区至少新谋划推进一个投资10亿元以上、三个5亿元以上基础设施项目。促进有能力在城镇稳定就业和生活的常住人口有序市民化。

　　（八）实施大规模国土绿化和“四水同治”行动，构建生态文明体系。大幅增绿、大兴水利，建设天蓝地绿、山清水秀的美丽新乡。

　　打造生态林网。按照“山区森林化、平原林网化、城市园林化、乡村林果化、廊道林荫化、庭院花园化”要求，编制实施森林新乡建设规划。把“32231”林业生态工程作为建设森林新乡的载体和抓手，将国家储备林资源向沿黄生态经济带、南太行生态景观带、卫河生态景观带、山水林田湖草一体化生态城、延津县主题森林城等重大项目倾斜。提升主要河道、过境高速、旅游通道等重要廊道两侧绿化标准。编制黄河湿地鸟类自然保护区和太行山猕猴自然保护区提升方案，启动生态提升项目建设。加快获嘉县、封丘县、凤泉区、牧野区苗木基地建设，力争育苗总面积达到5万亩，满足我市国土绿化苗木需求。

　　建设生态水网。推进“四水同治”，加快“五湖四河两渠”水生态体系建设，上半年南湖公园投入使用，卫源湖、平原湖（二期）取得明显进展。做好省十大水利工程涉及我市的西霞院水利枢纽输水及灌区工程、卫河共产主义渠治理、黄河下游贯孟堤扩建等项目前期工作。加快推进南水北调“四县一区”新增饮水工程。做好薄壁、石门河两处调蓄工程前期工作，争取项目尽早实施。高质量策划、规划、建设卫河综合治理工程，使之建成集水利河道、滨河绿道、景观廊道于一体的带状公园，形成卫河风情、运河文化与城市建设相得益彰的生态文化景观带。

　　（九）加强基础设施建设，构建基础能力支撑体系。弥补短板、提升优势，构建现代基础设施网络。

　　构建畅通路网。围绕郑新深度融合、市县融合、城乡融合，谋划推进重大交通基础设施项目。做好郑济铁路新乡段征地拆迁和施工环境保障等工作，做好新焦城际铁路前期工作和郑新市域铁路方案研究。谋划高铁东站双面站和零换乘中心建设，打造全省和郑州大都市区重要的铁路客货运枢纽。完成郑新快速通道和G230前期工作，促进郑新之间基础设施互联互通。加快“三条高速、三条公路”建设，新晋高速、新日高速封丘至原阳段均完成土石方工程量的70%；沿太行高速新乡段完成土石方工程量的50%；新延快速路9月底前竣工通车，打造成为市政化快速通道的样板工程；S229辉县段、凤泉段、新乡县段全面开工；G107东移改线按照合同约定，达到时序进度要求。加快推进中心城区与各城市组团的交通连接，建设市政化快速通道，推动一体化发展，力争新获、新卫、新新三条市域快速路和幸福渠穿滩公路、黄河大堤堤顶生态通道、沿黄西路三条沿黄公路建成通车。力争濮卫高速新乡段开工建设。加快官渡黄河大桥、菏宝高速长垣段等项目建设，保障好鹤辉高速项目建设环境。做好安罗高速新乡段、新日高速兰考至封丘段、郑新高速（新晋高速南延）、G230新乡黄河大桥等项目前期工作。抓好高速公路上下站口建设，做好京港澳高速新乡站、长济高速新乡西站改扩建工作，新建京港澳高速新乡新区站，尽快迁建京港澳高速原阳站。启动新乡高速公路和快速通道环线建设，争取新乡南环高速纳入全省高速公路网规划。

　　提速升级通信网。抓住数字化、网络化、智能化融合发展的重大机遇，抓好数字基础设施建设，加快骨干传送网扩容升级，大幅提高无线网、有线宽带接入能力。加快第五代移动通信网络建设和商用进程，促进工业互联网及自动化发展。全力支持中电科22所与国内外机构在物联网、人工智能等方面开展合作，加快中国电波科技城建设，加强天博物联网研究院创新能力建设。将华为新乡云计算数据中心打造成面向全国服务的“信息高速公路枢纽”，为新乡经济发展赋能，为市民幸福生活添彩。

　　提升能源保障能力。推进可再生能源开发利用，在风电、生物质能等领域新增发电装机15万千瓦。建设5项220千伏输变电工程，提升城乡电网保障能力。依托中石油西气东输豫北支线等燃气长输干线工程，加快配套支线管网建设。深入推进“温暖新乡守护蓝天”工程，啃下市区供热最后的“硬骨头”，基本实现全覆盖。

　　（十）保障和改善民生，构建公共服务体系。坚持以人民为中心的发展思想，尽力而为、量力而行，切实保障基本民生，推动解决重点民生问题，促进社会公平正义，让人民过上好日子。

　　抓好就业和社会保障工作。完善大学生留新就业创业政策，做好就业困难人员、退役军人等群体就业工作；完善城乡统一的居民养老保险、基本医疗保险等制度，做好社会保险扩面征缴，确保基本养老金按时足额发放。城市最低生活保障标准由每人每月不低于470元提高到不低于520元，农村最低生活保障标准由每人每年不低于3450元提高到不低于3860元。健全农村留守儿童、妇女、老人、残疾人医疗康复救助等服务体系。

　　大力发展教育事业。新建和改扩建幼儿园52所，新增学位8547个。两年内完成义务教育“改薄”、普惠幼儿教育、普及高中阶段教育任务。筹建新乡科技应用学院。加快新乡职业技术学院二期工程建设。

　　推进国际合作办学，支持驻新高校发展。

　　提升医疗卫生服务水平。深化公立医院改革，推进分级诊疗、家庭医生签约服务和紧密型医联体建设。加快中心医院东区医院、妇幼保健院等项目建设，谋划第四人民医院迁建工作，推进英国伯明翰大学医院在平原示范区建立国际合作医院。持续提升妇幼健康服务能力。参加城乡居民基本医保的80岁以上老人住院报销比例在现行政策基础上提高5个百分点，实施80岁以上老人高龄津贴制度。

　　推动文化体育事业发展。践行社会主义核心价值观，弘扬新乡先进群体精神。加强文物保护和传承利用，策划规划实施一批重大文化产业项目，加快大运河文化带建设规划编制，推进百泉景区保护开发。提升基层综合性文化服务中心功能，广泛开展文化惠民活动。实施全民健身计划，加快建设全国青少年校园足球改革试验区，加快平原体育中心等体育场馆建设，争取承办一批国际国内重大赛事。

　　加强社会治理创新。推进城乡社区管理规范化，加快社区新时代文明实践中心、社会治安综合治理中心、青少年活动中心、志愿服务中心、社区健身活动区、社区医院、便民超市建设，打造“六星示范社区”。解决存量问题楼盘85%以上，严防产生新的问题楼盘。完善信访工作机制，落实“一村一警”制度，完善诉调对接矛盾纠纷多元化解机制，解决好群众的合理诉求。严厉打击食品药品违法行为，强化农畜产品质量安全监管，抓好非洲猪瘟等疫病防控，争创国家食品安全示范城市。深入开展扫黑除恶专项斗争，打击各类违法犯罪活动，确保社会大局稳定。

　　统筹各项事业发展。支持驻新部队建设，深入开展双拥共建，完善退役军人服务保障体系，巩固军政军民团结。依法管理宗教事务，促进民族团结。加强统计工作，做好第四次经济普查。支持工会、共青团、妇联等人民团体更好发挥桥梁纽带作用，做好外事、海关、侨务、史志、档案、气象、地震、人防等各项工作。

　　三、加强政府自身建设，全面提高履职能力和服务水平

　　全力打造信念过硬、政治过硬、责任过硬、能力过硬、作风过硬的公务员队伍，建设亲民、法治、责任、实干、清廉政府。

　　（一）树牢“四个意识”，坚定“两个维护”。把学习贯彻习近平新时代中国特色社会主义思想往深里走、往实里走、往心里走，树牢“四个意识”、坚定“四个自信”、做到“两个维护”，在政治立场、政治方向、政治原则、政治道路上同以习近平同志为核心的党中央保持高度一致。严格遵守政治纪律和政治规矩，坚持把党对一切工作的领导贯穿到政府工作各领域、全过程，有令则行、有禁则止，不讲条件、不搞变通，确保中央和省委、省政府及市委各项决策部署落到实处。

　　（二）弘扬法治精神，推进依法行政。严格按照法定权限和法定程序行使权力，善于运用法治思维和法治方式深化改革、推动发展、化解矛盾、维护稳定。坚持依法决策，充分发扬民主，正确实行集中，提高决策科学化、民主化、法治化水平。推进政务公开，主动回应社会关切。自觉接受人大法律监督、工作监督和政协民主监督，强化审计监督，支持监察监督和司法监督，主动接受社会和舆论监督。推进严格规范公正文明执法，坚决防止任性用权，切实纠正粗暴执法，决不允许以权代法、以权压法、逐利违法、徇私枉法。

　　（三）提升能力素质，坚持从严治政。政府工作人员要加快知识更新，弥补知识弱项、能力短板，防止思想僵化、本领弱化。当官避事平生耻，视死如归社稷心。要发扬斗争精神，提升斗争本领，砥砺斗争意志，敢于在困难面前挺身而出、横刀立马、斩关夺隘、敢打必胜，在推进重点工作、解决重大矛盾、完成重要任务中增强学习思考本领、改革创新本领、驾驭风险本领、依法执政本领、群众工作本领。关心基层干部，为基层减负，为担当者担当、为负责者负责。落实政府系统全面从严治党主体责任，持之以恒整治“四风”，扎牢织密制度笼子，铲除滋生腐败土壤，坚决查处腐败行为。

　　（四）健全完善机制，确保工作落实。做到推进工作机制化，建立任务清单、问题清单、责任清单和专案专人专班专责的“三单四专一台账”机制，把目标任务分解到部门、具体到项目、落实到岗位。政府工作不能驰于空想、骛于虚声、信马由缰，要以抓落实为核心，形成议事有规则、管理有办法、操作有程序、过程有监控、责任有追究的推进格局，构建权责明晰、运转高效、衔接严密的重点工作运转体系。

　　各位代表！伟大梦想不是等得来、喊得来的，而是拼出来、干出来的。让我们紧密团结在以习近平同志为核心的党中央周围，高举中国特色社会主义伟大旗帜，以奋发有为的状态、敢闯敢试的斗志、开拓进取的精神，全力推动经济社会高质量发展，为决胜全面建成小康社会、建设富强民主文明和谐美丽的社会主义现代化强市而努力奋斗，以优异成绩迎接中华人民共和国成立70周年！
